# Supplementary material for: Unique structure of active platinum-bismuth site for oxidation of carbon monoxide
Source: Nat Commun. 2021 Jun 7;12:3342. doi: 10.1038/s41467-021-23696-7 (PMC8184822; doi:10.1038/s41467-021-23696-7)
Supplement: Supplementary file 1 — Supplementary Information [file 41467_2021_23696_MOESM1_ESM.pdf]

Supplementary Materials for

Unique structure of active platinum-bismuth site for oxidation of carbon monoxide

Bing Nan,[1, 2] Qiang Fu,[3] Jing Yu,[4] Miao Shu,[1] Lu-Lu Zhou,[3] Jinying Li,[3] Wei-Wei Wang,[3] Chun-Jiang Jia,[3]* Chao Ma,[5]* Jun-Xiang Chen,[6] Lina Li,[1, 7] Rui Si,[1, 7]*
*Correspondence to: Email: sirui@sinap.ac.cn (R.S.); jiacj@sdu.edu.cn (C.-J.J.); cma@hnu.edu.cn (C.M.)

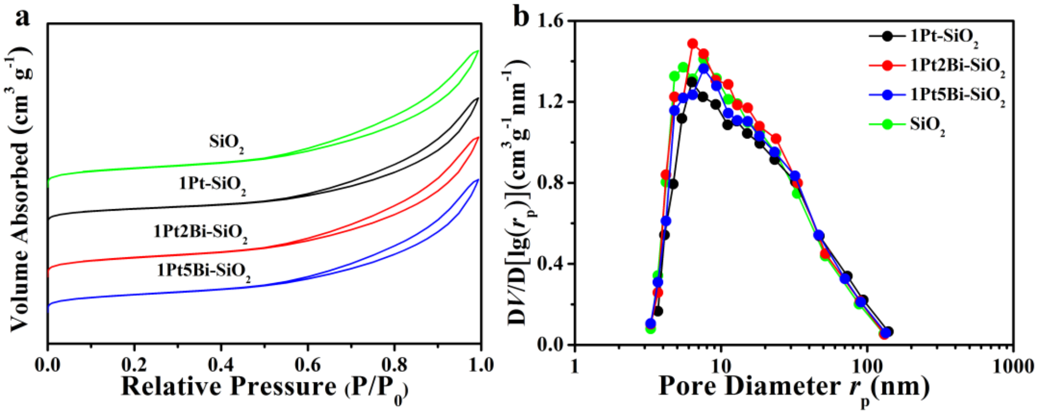

Supplementary Fig. 1 Nitrogen adsorption measurements of fresh Pt/PtBi-SiO_2 samples: (a) adsorption-desorption isotherm and (b) BJH pore size distribution.

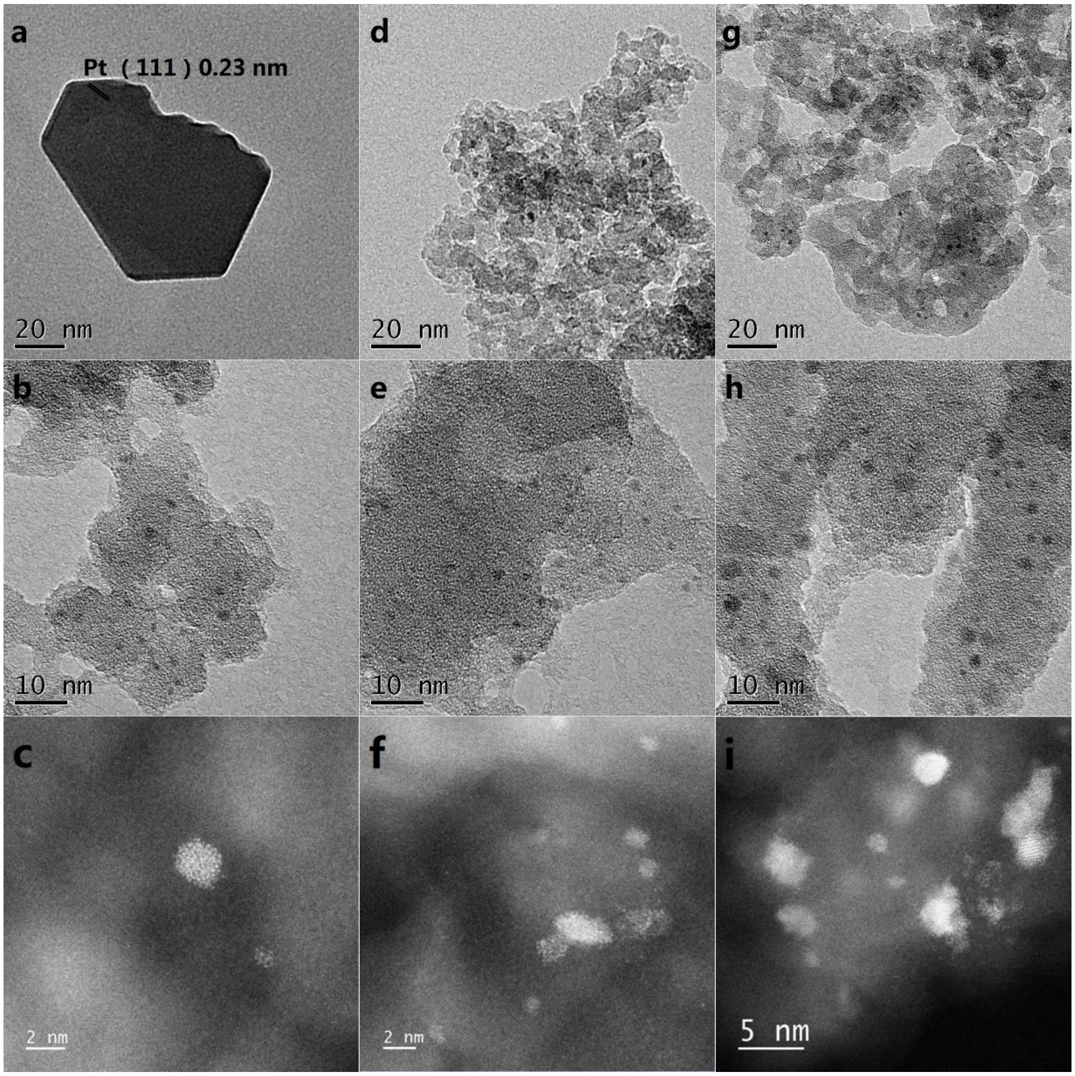

Supplementary Fig. 2 TEM (a,d,g), HRTEM (b,e,h) and aberration-corrected HAADF-STEM (c,f,i) images of fresh Pt/PtBi-SiO_2 samples: (a,b,c) 1Pt-SiO_2; (d,e,f) 1Pt2Bi-SiO_2; (g,h,i) 1Pt5Bi-SiO_2.

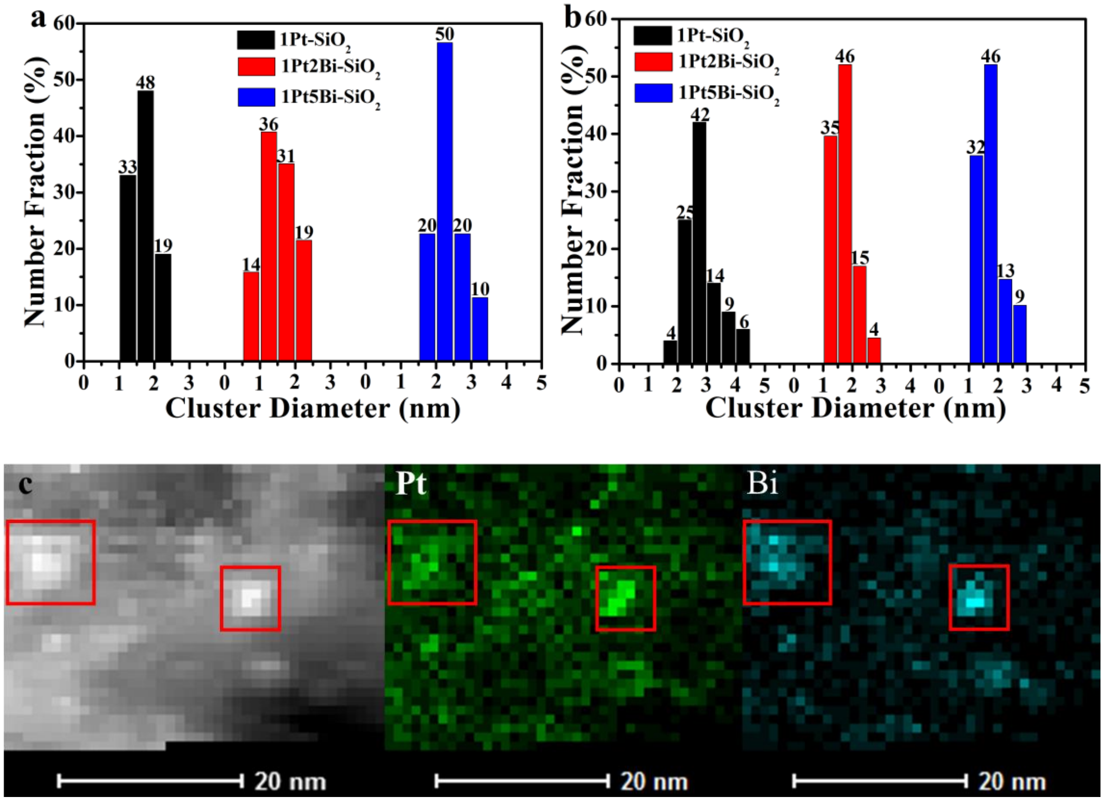

Supplementary Fig. 3. Diameter histograms on oxide cluster of fresh (a) and used (b) Pt/PtBi-SiO_2 samples on the basis of HAADF-STEM data and (c) the STEM-EDS mapping image of fresh 1Pt2Bi-SiO_2 of single oxide cluster.

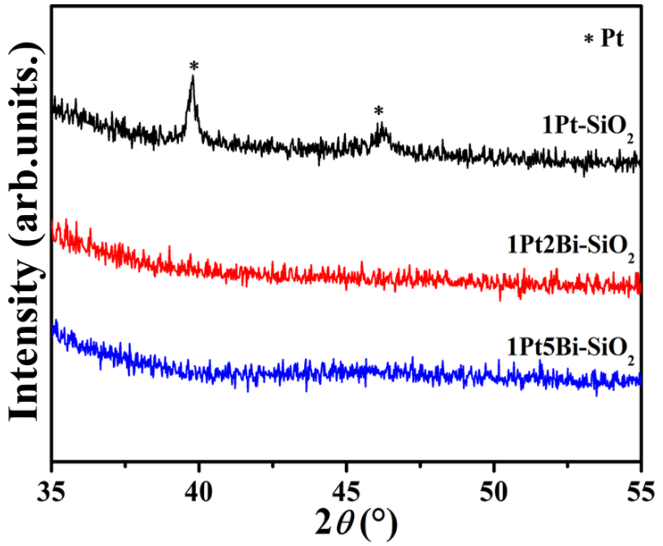

Supplementary Fig. 4 The XRD patterns of fresh Pt/PtBi-SiO_2 samples with a "slow-scan" mode (scanning rate of 1° min^{-1}) from 35 to 55°.

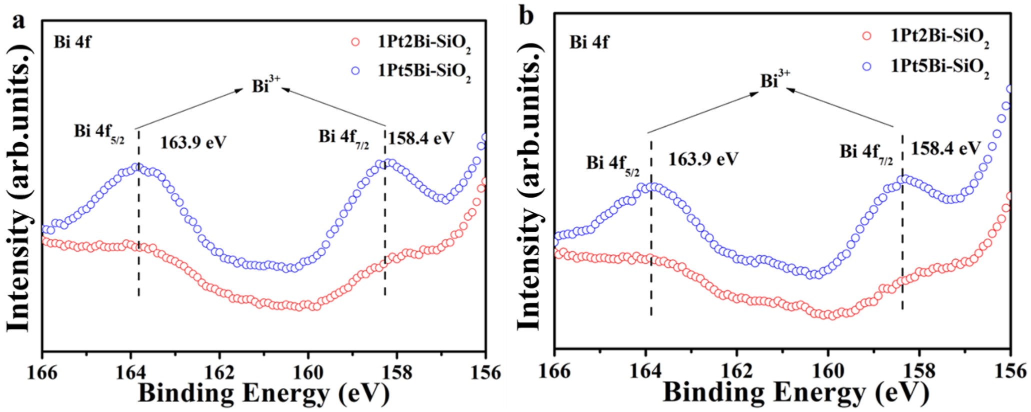

Supplementary Fig. 5 X-ray photoelectron spectroscopy profiles of PtBi-SiO_2 samples: (a) fresh; (b) used.

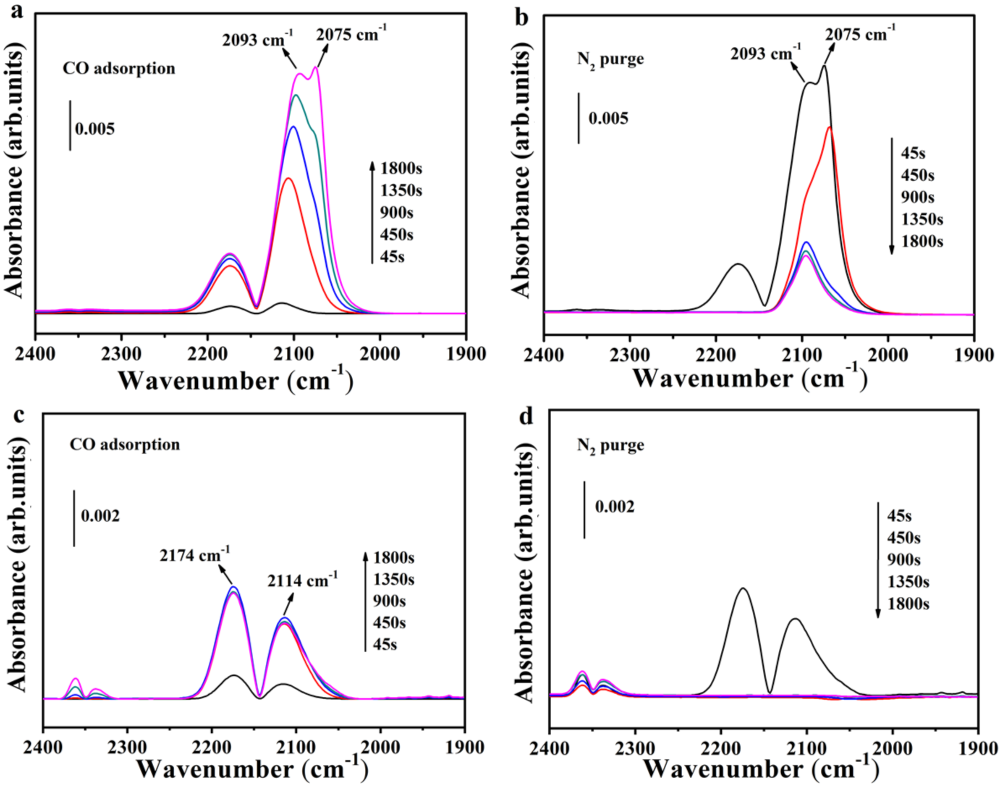

Supplementary Fig. 6 In-situ DRIFTS study of (a,c) CO adsorption, (b,d) N_2 purging on fresh 1Pt-SiO_2-400 (a,b) and 1Pt2Bi-SiO_2 (c,d). The catalysts were pretreated in situ at 300 °C under O_2 flow in the DRIFTS reaction cell before data collection (CO flow rate: 30 mL min^{-1}; catalyst mass: 20 mg; temperature: 100 °C)

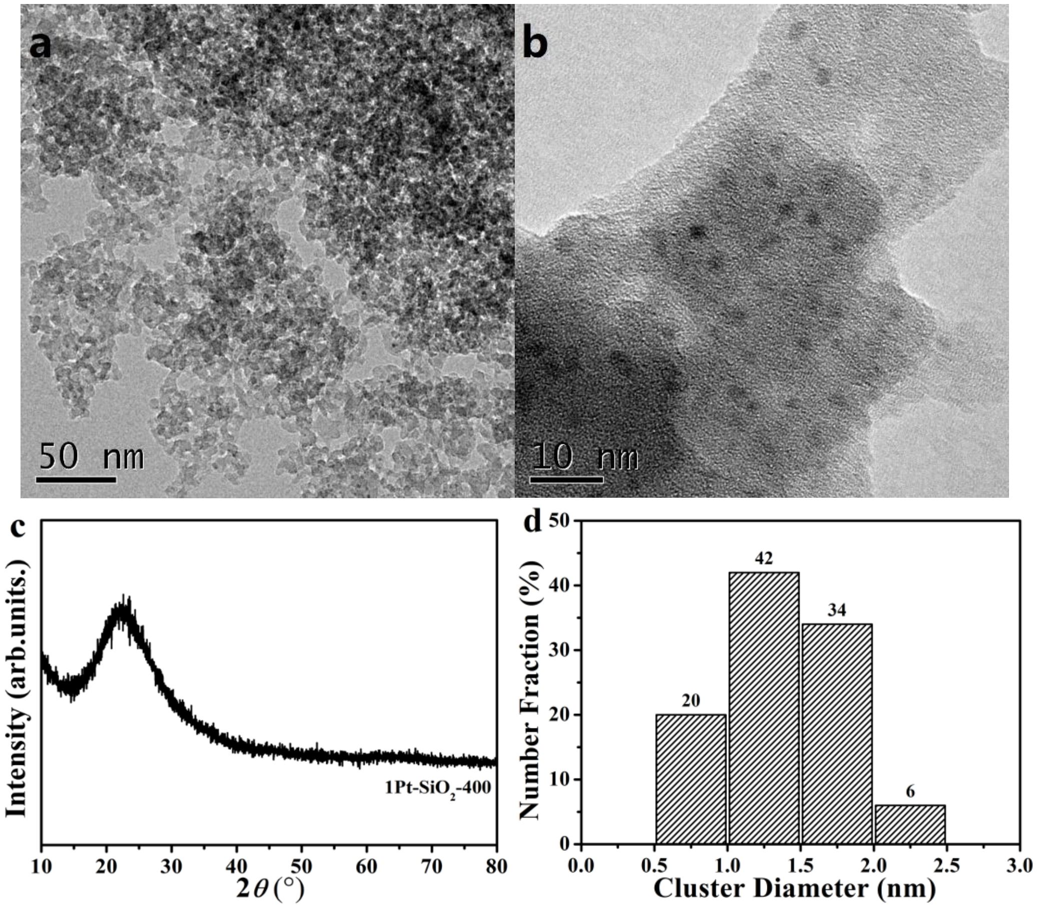

Supplementary Fig. 7 TEM/HRTEM (a,b) images; XRD profile (c) and diameter histograms on oxide clusters (d) for fresh 1Pt-SiO_2-400.

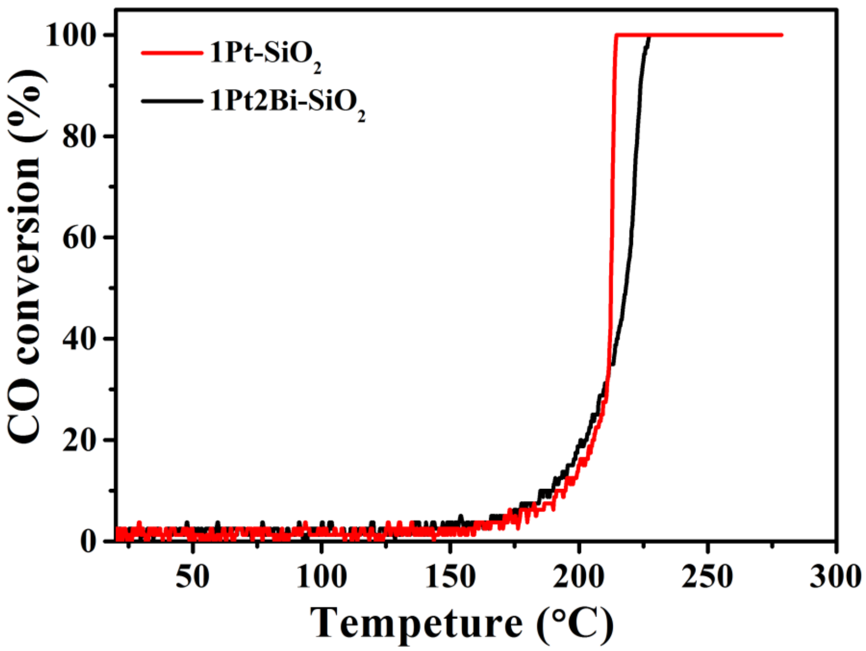

Supplementary Fig. 8 CO oxidation light-off experiments for Pt/PtBi-SiO_2 samples with pretreatment at 300 °C under air (1% CO/20% O_2/79% N_2, 134,000 mL g_{cat}^{-1} h^{-1}).

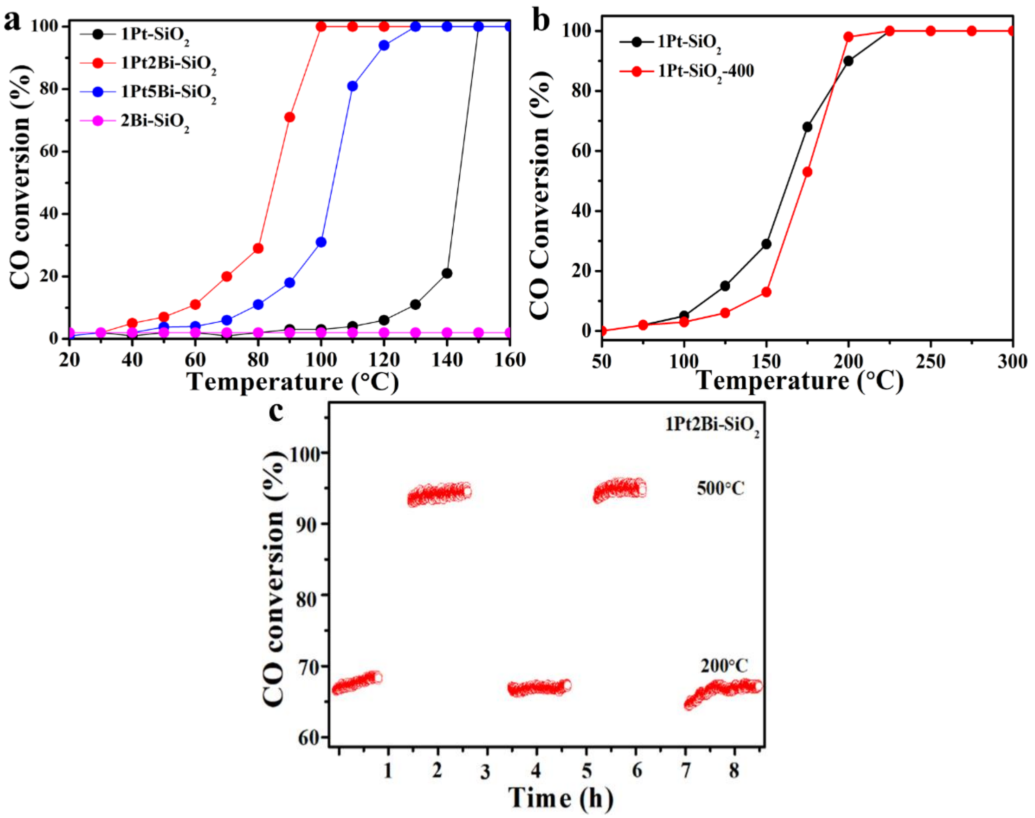

Supplementary Fig. 9 (a) the "light-off" experiments over Pt/PtBi-SiO_2 samples; (b) the catalytic performance of 1Pt-SiO_2 and 1Pt-SiO_2-400; and (c) the cyclic stability of the 1Pt2Bi-SiO_2 alternatingly tested at 500 and 200 °C (2% CO/1% O_2/He, 10 mg of catalyst, GHSV: 300, 000 mL·gcat^{-1}·h^{-1})

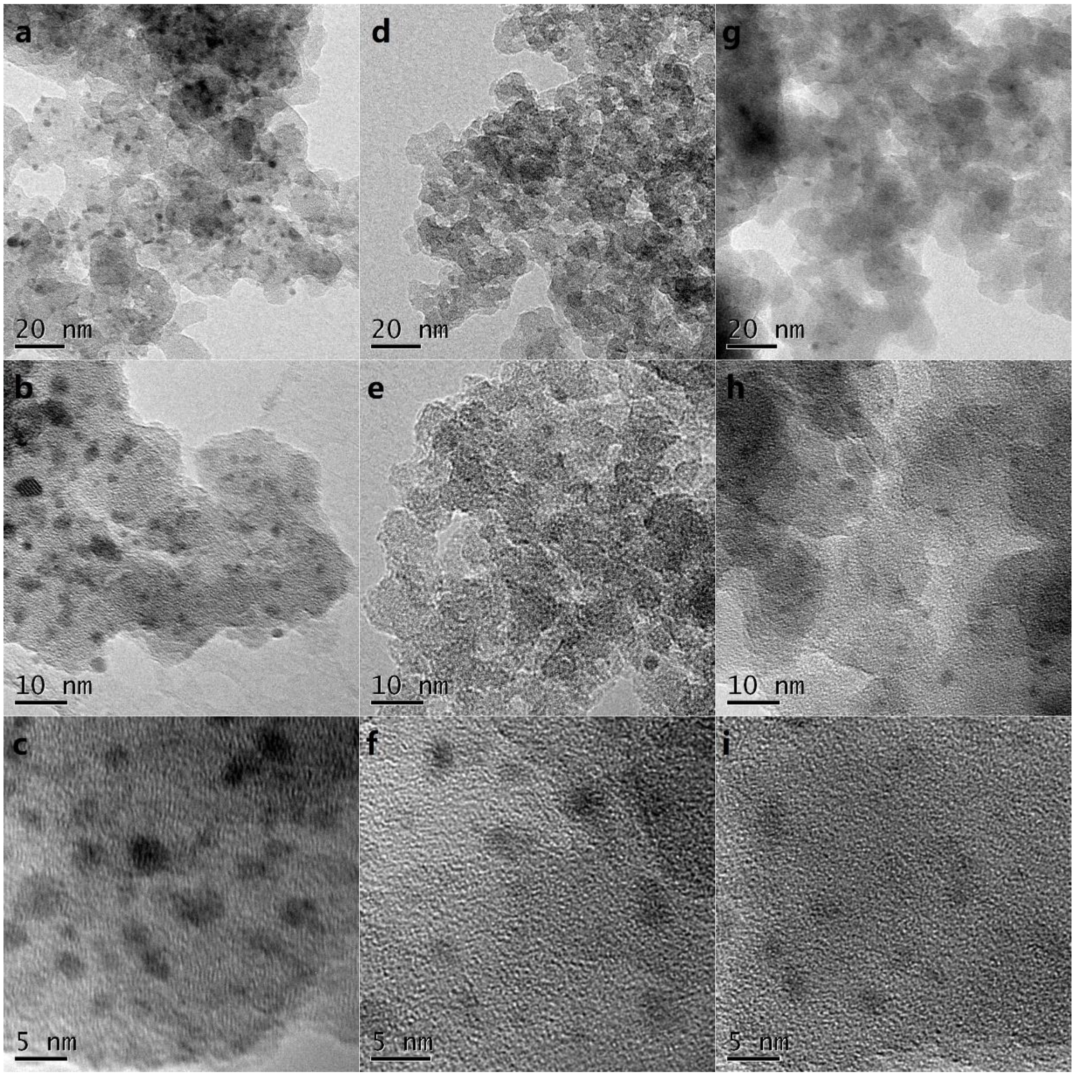

Supplementary Fig. 10 TEM and HRTEM images of used Pt/PtBi-SiO_2 samples: (a,b,c) 1Pt-SiO_2; (d,e,f) 1Pt2Bi-SiO_2; (g,h,i) 1Pt5Bi-SiO_2.

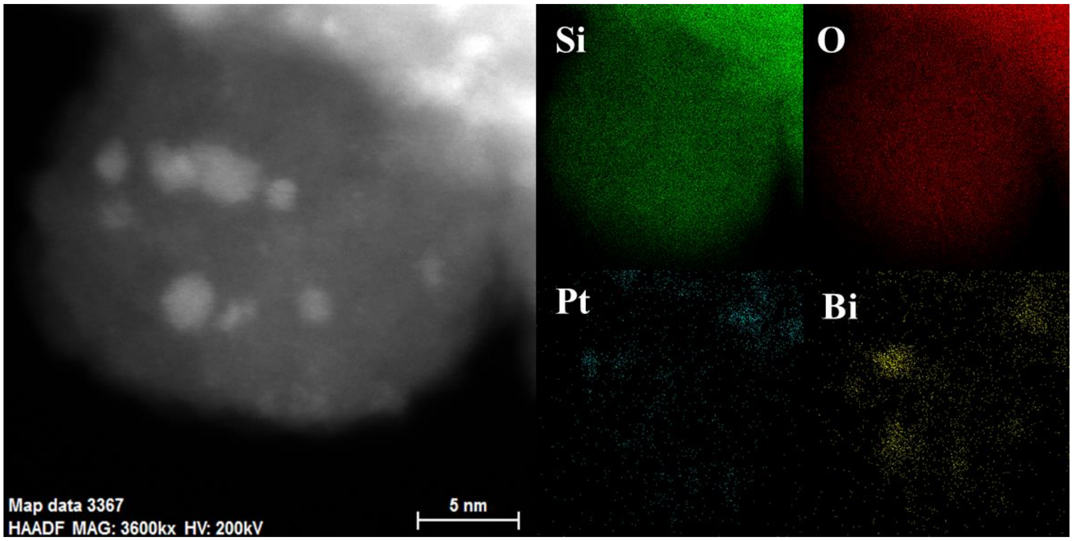

Supplementary Fig. 11 Representative aberration-corrected STEM-EDS elemental mapping images of used 1Pt2Bi-SiO_2.

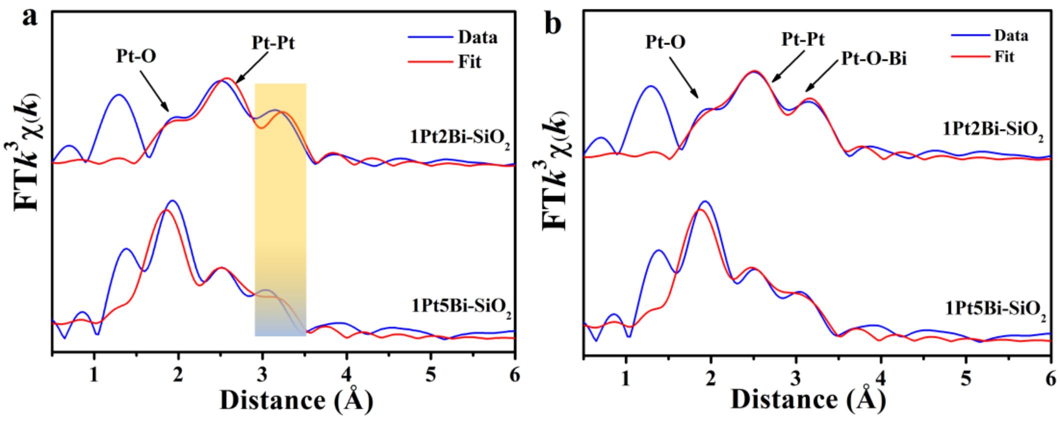

Supplementary Fig. 12 The EXAFS fitting results in *R* space of PtBi-SiO_2 samples after hydrogen reduction at 210 °C for 30 mins without air exposure until finishing XAFS experiments: (a) only Pt–O and metallic Pt–Pt shells and (b) Pt–O, metallic Pt–Pt and Pt–O–Bi shells for EXAFS fitting.

We have conducted the XAFS tests for Bi-doped samples after hydrogen reduction at 210 °C without air exposure, well consistent with the pretreatment condition before CO oxidation "light off" experiment to detect structural evolution of active site. Firstly, we supposed that the hydrogen reduction destroyed Pt−$[O]_x$−Bi structure completely with metallic Pt−Pt shell (small-size metallic Pt clusters or particles) plus a minor Pt−O shell. However, the fitting results with only Pt−Pt and Pt−O shells in Supplementary Fig. 12a were not well consistent with EXAFS data, especially in ~3.0 Å lacking a specific shell. Therefore, we appended an additional Pt−O−Bi shell for Bi-promoted samples, in which the fitting curves could match EXAFS data pretty well. It indicated that after hydrogen reduction the active site was composed of metallic Pt phase plus Pt−$[O]_x$−Bi structure.

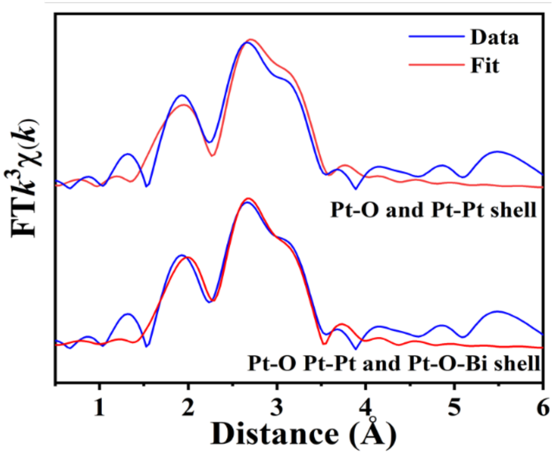

Supplementary Fig. 13 The comparison of different shells over EXAFS fitting results in *R* space for 1Pt2Bi-SiO_2.

In order to require more reliable local structure for used Bi-promoted samples, we firstly conducted the EXAFS fitting process with Pt–O and metallic Pt–Pt shells. Although we can obtain the relatively reasonable coordination number and bond distance for Pt–O and metallic Pt–Pt shells, the fitting curves were not consistent with EXAFS profiles very well, especially in 3.0–3.5 Å. After addition of Pt–O–Bi shell, we can acquire the most suitable EXAFS fitting results in Supplementary Fig. 13.

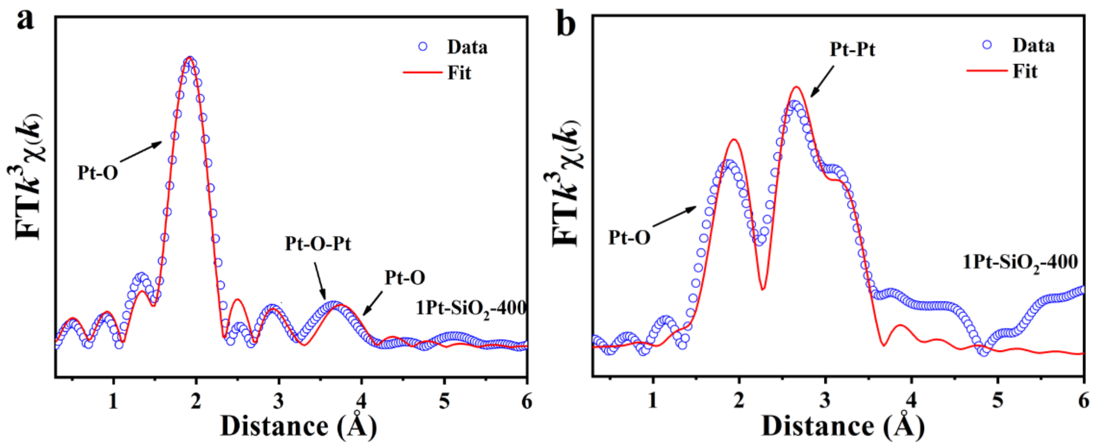

Supplementary Fig. 14 Pt L_3–edge EXAFS fitting results in *R* space for (a) fresh and (b) used 1Pt-SiO_2-400.

As a reference, 1Pt-SiO_2-400 calcinated at 400 °C possesses almost identical local coordination structure and active site size with Bi-promoted samples except for Pt–$[O]_x$–Bi structure. After CO oxidation, the Pt active site is in a low oxidized state for 1Pt-SiO_2-400 (+0.9) sample and EXAFS results indicate the Pt–O ($R \approx 2.00$ Å, $CN \approx 2.0$) and metallic Pt–Pt ($R \approx 2.75$ Å, $CN \approx 5.8$) shell about ~1 nm nanoparticle with surface partial oxide platinum site.

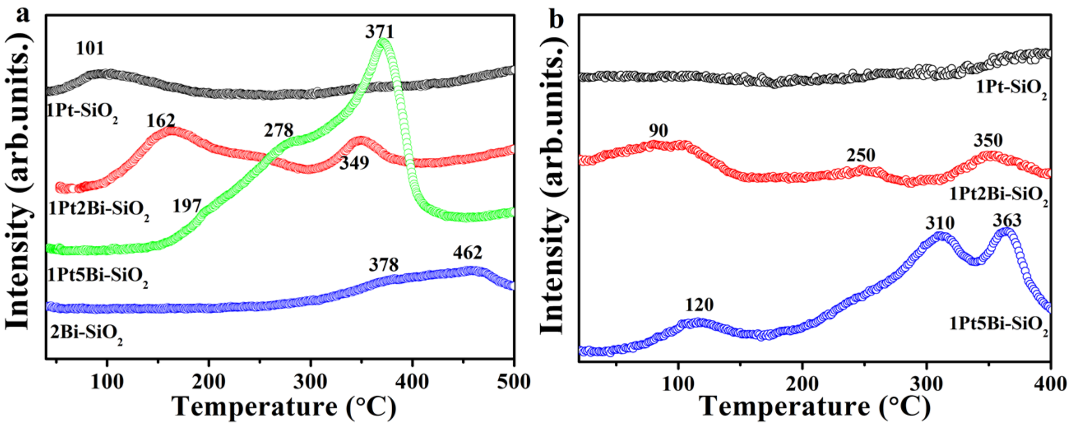

Supplementary Fig. 15 H_2–TPR profiles of fresh (a) and used (b) Pt/PtBi-SiO_2 catalysts. The H_2-TPR for used samples was conducted after CO oxidation without exposing to air.

For used samples, the reducible oxygen in 1Pt-SiO_2 has been taken away by hydrogen pretreatment at 210 °C, being metallic platinum particle. However, a low temperature reduction peaks for used Bi-promoted samples further confirmed the maintaining of Pt−[O]$_x$–Bi structure. We calculated the hydrogen consumption of 1Pt2Bi-SiO_2 (78μmol/g) and 1Pt5Bi-SiO_2 (95μmol/g) for Pt−[O]$_x$–Bi structure, which was much less than that of fresh Bi-promoted samples. It indicated that the most Pt−[O]$_x$–Bi structure was destroyed. Moreover, no temperature shift occurred on peaks for isolated BiO_x cluster (300−400 °C), revealing that BiO_x cluster structure could be maintained after hydrogen reduction and make no contribution on improvement of CO oxidation activity.

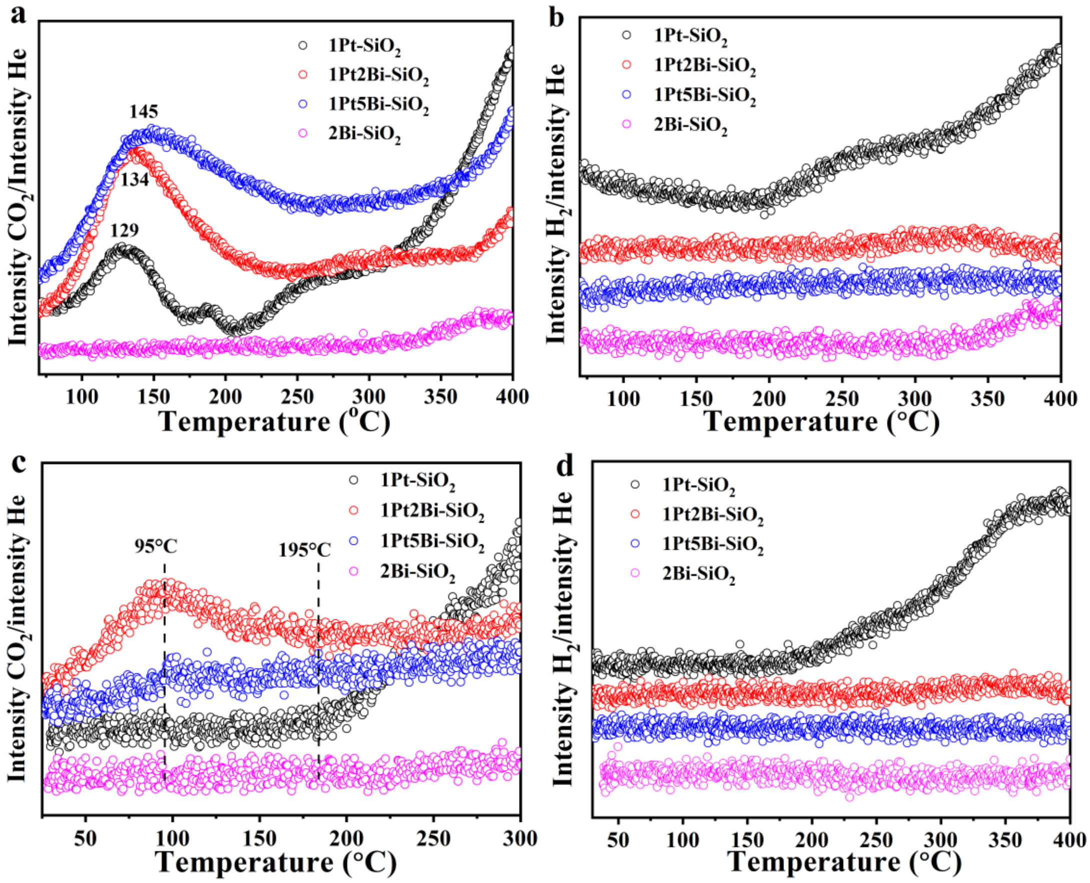

Supplementary Fig. 16 CO–TPR profiles of (a,b) fresh (pretreatment condition: 300 ºC for 30 min in 5 vol.% O_2/He) and (c,d) used (after CO oxidation without air exposure) Pt/PtBi-SiO_2 samples, (5 vol.% CO/He, 20 cm^3 min^{-1}, with ramping rate at 5 °C min^{-1})

The amount of surface oxygen was calculated by following formula:

Active oxygen quantity: $R = (V \times 0.05 \times Con.) / (22.4 \times M) \times 1000 \times t$ (1)

V: gas flow rate Con.: conversion of 5%CO/He M: mass of catalyst t: time of first peak for CO-TPR 0.05: 5%CO/He

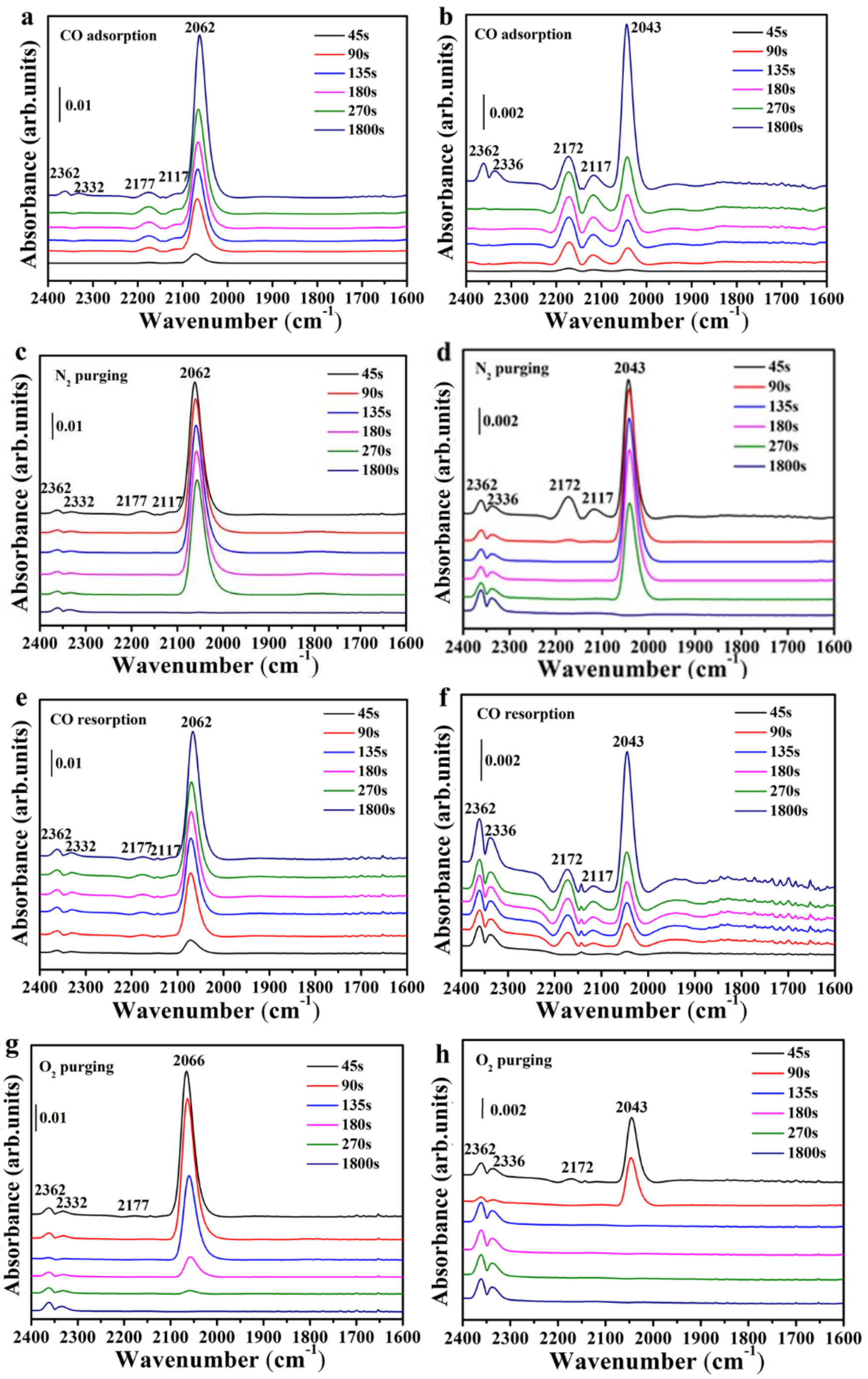

Supplementary Fig. 17 In-situ DRIFTS study of (a, b) CO adsorption, (c, d) N_2 purging, (e, f) CO resorption, and (g, h) O_2 removal on (a, c, e, g) 1Pt-SiO_2 and (b, d, f, h) 1Pt2Bi-SiO_2. The catalysts were pretreated in situ at 210 °C under 5 vol.% H_2/N_2 flow in the DRIFTS reaction cell before data collection (CO flow rate: 30 mL min^{-1}; catalyst mass: 20 mg; temperature: 100 °C).

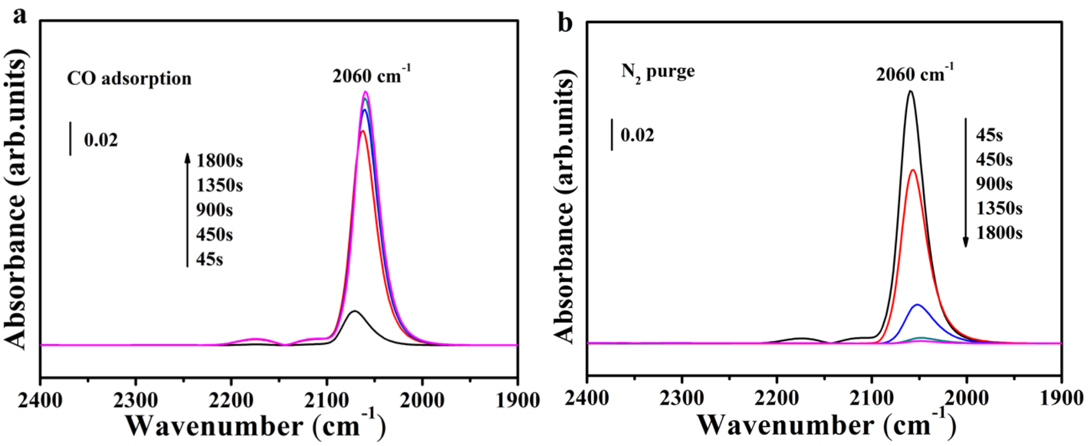

Supplementary Fig. 18 In-situ DRIFTS study of (a) CO adsorption, (b) N_2 purging on 1Pt-SiO_2-400. The catalysts were pretreated in situ at 210 °C under 5 vol.% H_2/N_2 flow in the DRIFTS reaction cell before data collection (CO flow rate: 30 mL min^{-1}; catalyst mass: 20 mg; temperature: 100 °C)

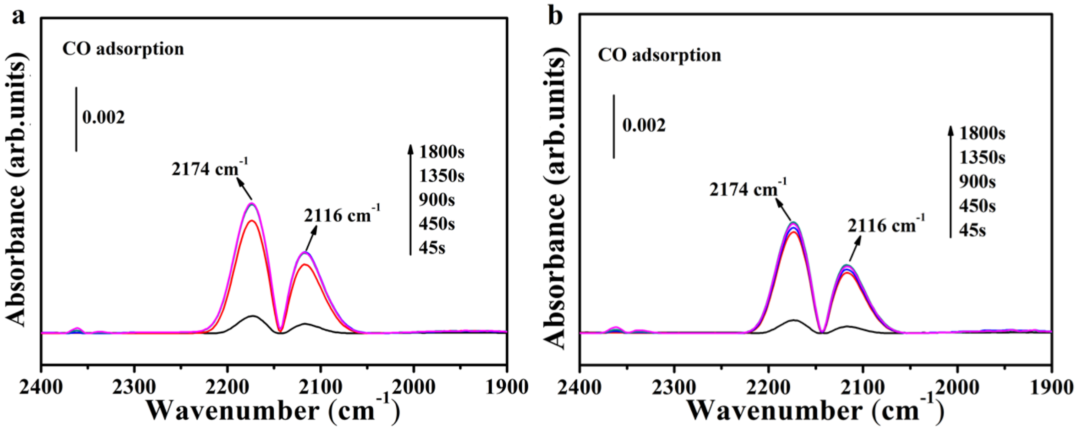

Supplementary Fig. 19 In-situ DRIFTS study of CO adsorption on 2Bi-SiO_2 with different pretreatment condition. The catalysts were pretreated in situ (a) at 300 °C under air and (b) at 210 °C under 5 vol.% H_2/N_2 flow in DRIFTS reaction cell before data collection (CO flow rate: 30 mL min^{-1}; catalyst mass: 20 mg; temperature: 100 °C)

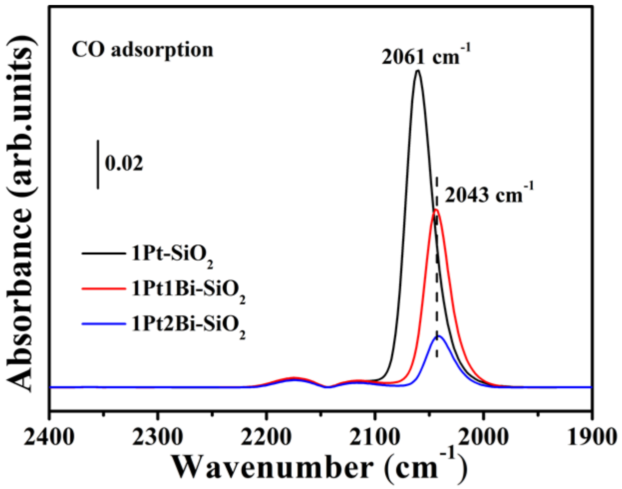

Supplementary Fig. 20 In-situ DRIFTS study of CO absorption on different doping of bismuth species. (CO flow rate: 30 mL min^{-1}; catalyst mass: 20 mg; temperature: 100 °C).

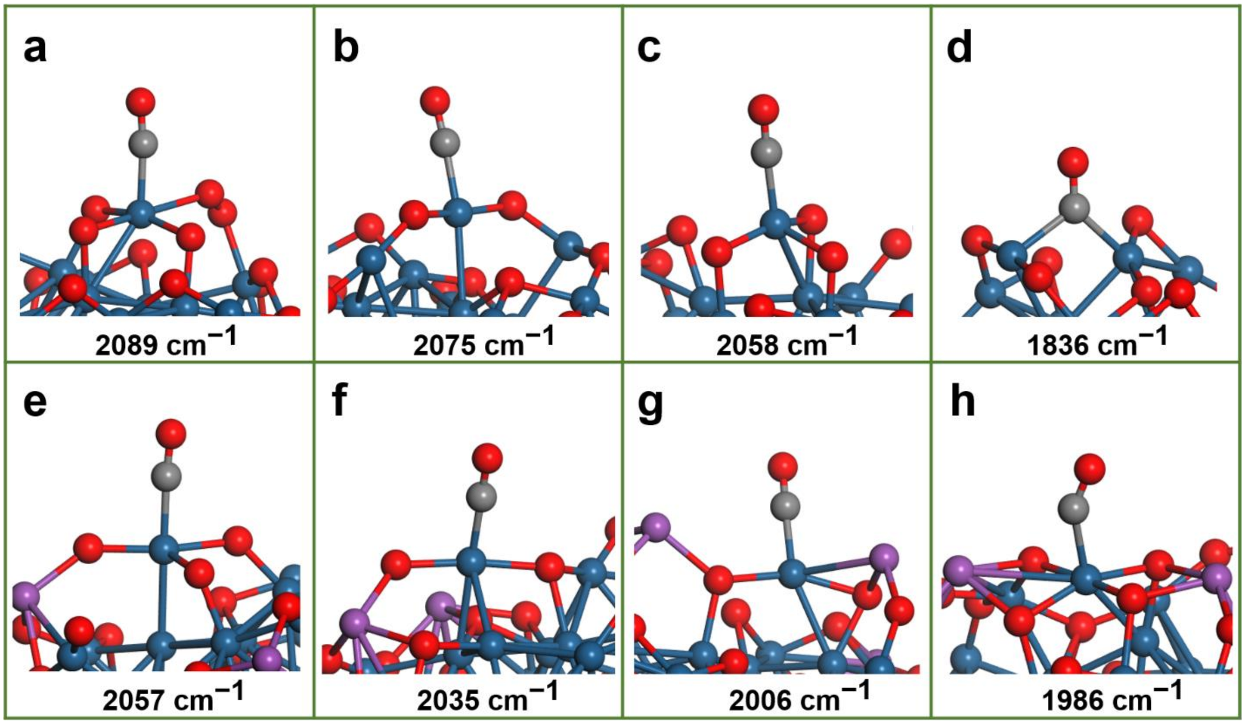

Supplementary Fig. 21 Frequencies of a CO adsorbate on the Pt@PtO$_x$ model without (a-d) and with (e-h) Bi dopant. The teal blue, gray, red and purple spheres represent Pt, C, O, and Bi atoms, respectively.

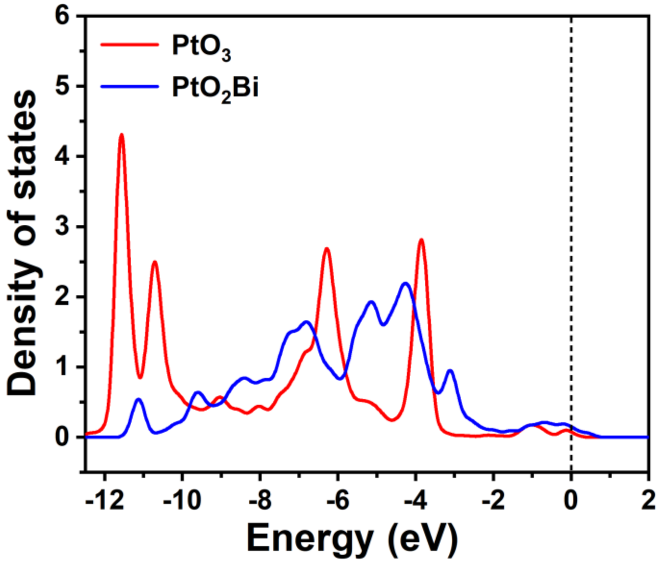

Supplementary Fig. 22 Density of states (DOS) for the *d* electrons of the Pt atom on which the CO molecule adsorbs. The red and blue lines correspond to the models in Supplementary Figs. 20c (without Bi atom) and 20f (with Bi atom), respectively. The Fermi level is set to 0 (black dash line).

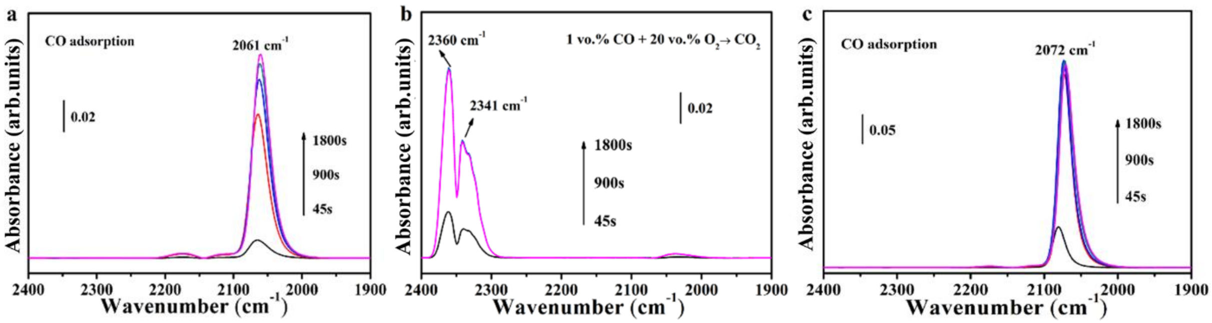

Supplementary Fig. 23 In-situ DRIFTS in the mode: "CO adsorption (a) → reaction conditions (1% CO/ 20% O_2/N_2 flow) at 200°C (b) → CO adsorption (c)" on 1Pt-SiO_2 without catalyst replacement and air exposure, (CO flow rate: 30 mL min^{-1}; catalyst mass: 20 mg).

Supplementary Table 1: Bulk Pt and Bi concentration, BET specific surface areas (S_{BET}), BJH pore volume (V_p), average cluster sizes (D) for Pt/PtBi-SiO_2 and SiO_2 samples.

Sample	Pt (wt.%)[a]	Bi (wt.%)[a]	T_R (°C)	H_2 (μmol g^{-1})[b]	S_{BET} (m^2/g)[c]	V_p (cm^3/g)[c]	D (nm)[d]
SiO_2	—	—	—	—	430	1.322	—
1Pt-SiO_2	0.8	—	101	157^{α}, 82^{β}	400	1.225	1.7±0.4<br>3.0±0.6[e]
1Pt2Bi-SiO_2	0.9	2.3	162, 248, 349	254^{α}, 247^{β}	424	1.352	1.6±0.5<br>1.8±0.3[e]
1Pt5Bi-SiO_2	0.9	6.1	197, 278, 371	657^{α}, 529^{β}	405	1.286	1.7±0.3<br>2.0±0.4[e]

[a] Determined by ICP-AES; [b] Actual value of H_2 consumptions (α) and theoretical values of H_2 consumption calculated according to $Pt^{4+} \rightarrow Pt^0$ and $Bi^{3+} \rightarrow Bi^0$ (β); [c] From N_2 adsorption/desorption experiments; [d] Statistic data of cluster on the basis of TEM images; [e] after CO oxidation.

Supplementary Table 2: The calculation formula for energy of Pt species stabilizing under different conditions

$$\Delta E_{average} = \left[E\left(Pt_x Bi_y O_z\right) - \left(x \cdot E(Pt) + y \cdot E(Bi) + z \cdot E(O_2)/2\right)\right]/x$$

We use $\Delta E_{average}$ as an indicator to describe the degree to which the Pt species is stabilized under different conditions. Where $E\left(Pt_x Bi_y O_z\right)$ is the total energy of the whole system, $E(Pt)$, $E(Bi)$, and $E(O_2)$ is the energy of a Pt atom, a Bi atom, and an O_2 molecule, respectively. It is worth noting that here the denominator only contains the number of Pt atoms, because we regard Pt as the object of existence, while O_2 and Bi are merely the environment in which the Pt species exists.

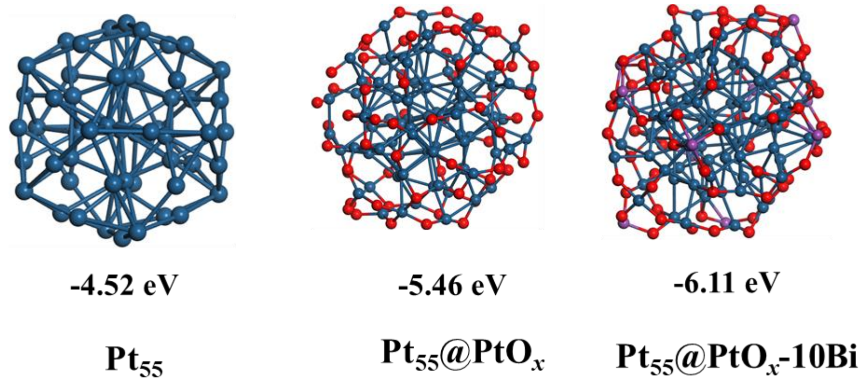

We have considered three systems, $Pt_{55}@PtO_x$-10Bi, $Pt_{55}@PtO_x$, and Pt_{55}, for the comparison of the $\Delta E_{average}$ value. Here, the PtO_x shell contains 32 Pt atoms and 66 O atoms in the simulation model. Our results show that the corresponding value for $Pt_{55}@PtO_x$-10Bi, $Pt_{55}@PtO_x$, and Pt_{55} is calculated to be -6.11 eV, -5.46 eV, and -4.52 eV, respectively. The large difference in the energy value per Pt atom indicates that the Pt atoms are stabilized the most when the O_2 atmosphere and the Bi dopants both exist. It thus supports the conclusion from the experiments that Bi can prevent the formation of Pt particles, as in this case, the Pt species will form bonds with O and Bi atoms at the same time.

Supplementary Table 3: Oxidation state of platinum (δ) and Pt L_3-edge EXAFS fitting results (R: distance; CN: coordination number; σ^2: Debye-Waller factor [a]; $\triangle E_0$: inner potential correction [b]) of Pt/PtBi-SiO_2 samples.

Sample	δ	Pt-O		Pt-Pt(metal)		Pt-O-Pt/Bi	
		R (Å)	CN	R (Å)	CN	R (Å)	CN
1Pt-SiO_2 (fresh)	1.8	2.01±0.01	2.4±0.3	2.78±0.01	6.4±0.8	3.09±0.02	2.7±1.0
1Pt-SiO_2-400 (fresh)	3.2	2.00±0.01<br>3.78±0.03	4.5±0.2<br>2.4±1.0	—	—	3.10±0.04	1.2±0.6
1Pt2Bi-SiO_2 (fresh)	3.0	2.00±0.01<br>3.77±0.04	4.9±0.1<br>2.4±1.7	—	—	3.03±0.02	3.0±0.7
1Pt5Bi-SiO_2 (fresh)	3.5	2.00±0.01<br>3.80±0.03	5.7±0.3<br>1.7±0.7	—	—	3.05±0.04	4.0±1.0
1Pt2Bi-SiO_2 (hydrogen[c])	0.2	2.06±0.03	0.6±0.2	2.73±0.01	6.8±1.4	2.90±0.04	2.6±1.2
1Pt5Bi-SiO_2 (hydrogen[c])	0.4	2.00±0.01	2.0±0.2	2.72±0.01	5.0±1.2	2.90±0.04	1.4±0.9
1Pt-SiO_2 (used)	0.8	—	—	2.76±0.01	9.4±1.0	—	—
1Pt-SiO_2-400 (used)	0.9	1.97±0.01	2.0±0.2	2.75±0.01	5.8±0.5	—	—
1Pt2Bi-SiO_2 (used)	1.3	1.99±0.01	1.0±0.1	2.77±0.01	8.4±1.7	2.99±0.02	2.2±0.8
1Pt5Bi-SiO_2 (used)	2.0	1.99±0.01	2.5±0.1	2.70±0.01	4.7±1.0	2.96±0.03	2.2±0.6

[a]σ^2 = 0.0030, 0.008 and 0.008Å^2 for all the analyzed Pt–O, Pt–Pt and Pt–Bi shells, respectively; [b]$\triangle E_0$ = 11.2, 13.3 and 14.1 eV for fresh 1Pt-SiO_2, 1Pt2Bi-SiO_2 and 1Pt5Bi-SiO_2, respectively, which were obtained from the linear combination fits on XANES profiles and the fitting results of Pt foil ($\triangle E_0$ = 8.3 ± 1.2 eV) and PtO_2 ($\triangle E_0$ = 15.0 ± 0.9 eV) standards; $\triangle E_0$ = 7.4 ± 1.8 eV for all used samples; [c] the samples was reduced with 5 vol. %H_2/N_2 at 210 °C for 30 min.

Supplementary Table 4: Rates normalized by catalyst weight (r_w), rates normalized by platinum amount (r_{Pt}) and apparent activation energies (E_a) for the carbon monoxide oxidation reaction over Pt/PtBi-SiO_2 samples.

Sample	r_w ($\mu mol_{CO} \cdot g_{cat}^{-1} \cdot s^{-1}$)			r_{Pt} ($mmol_{CO} \cdot mol_{Pt}^{-1} \cdot s^{-1}$)			E_a(kJ/mol)
	90°C	100°C	110°C	90°C	100°C	110°C	
1Pt-SiO_2	0.2	0.3	0.7	4.8	7.3	17.1	70±4
1Pt-SiO_2-400	0.2	0.3	0.4	4.8	7.3	9.8	81±5
1Pt2Bi-SiO_2	1.7	3.1	3.9	41.4	75.5	95.1	52±4
1Pt5Bi-SiO_2	0.9	1.5	2.2	21.9	36.6	53.6	56±3

Supplementary Table 5: Oxidation state of platinum (δ) and Pt L_3-edge EXAFS fitting results (R: distance; CN: coordination number; σ^2: Debye-Waller factor [a]; $\triangle E_0$: inner potential correction [b]) of Pt/PtBi-SiO_2 samples.

Sample	δ	Pt-O		Pt-Pt(metal)		Pt-O-Bi	
		R (Å)	CN	R (Å)	CN	R (Å)	CN
1Pt-SiO_2-150H_2	1.2	2.00±0.01	0.6±0.1	2.74±0.02	7.0±0.3	—	—
1Pt-SiO_2-210H_2	0.2	—	—	2.75±0.02	9.0±0.4	—	—
1Pt-SiO_2-CO oxidation	1.0	—	—	2.75±0.02	9.2±0.5	—	—
1Pt2Bi-SiO_2-150H_2	2.5	1.98±0.01<br>3.64±0.04	2.1±0.2<br>1.9±1.0	2.75±0.01	4.8±1.1	2.98±0.04	1.8±1.0
1Pt2Bi-SiO_2-210H_2	0.4	1.98±0.02	0.6±0.2	2.75±0.01	7.4±1.0	2.90±0.03	2.1±1.3
1Pt2Bi-SiO_2-CO oxidation	1.3	2.00±0.02	1.5±0.3	2.74±0.01	7.0±1.1	2.90±0.03	2.3±1.1

[a]σ^2 = 0.003±0.001, 0.007±0.001 and 0.007±0.001 Å^2 for all the analyzed Pt−O, Pt−Pt and Pt−Bi shells, respectively; [b]$\triangle E_0$ = 6.7±0.4 for all 1Pt-SiO_2 samples

Supplementary Table 6: H_2-TPR peak temperature (T_R), and experiment hydrogen consumption (H_2) over Pt/PtBi-SiO_2 samples.

Sample	T_R (°C)		H_2 (μmol g^{-1}) for first peak	
	fresh	used	fresh	used
1Pt-SiO_2	101	–	107	0
1Pt2Bi-SiO_2	162, 248, 349	90, 250, 349	185	78
1Pt5Bi-SiO_2	197, 278, 371	120, 310, 363	199	95
